# A Rare Gastrointestinal Manifestation of Cryoglobulinemic Vasculitis: A Case Report

**DOI:** 10.7759/cureus.81418

**Published:** 2025-03-29

**Authors:** Linda Gavric, Muhammad Tayyab Tahir, Shani M Abraham

**Affiliations:** 1 Internal Medicine, Lake Erie College of Osteopathic Medicine, Bradenton, USA; 2 Internal Medicine/Nephrology, AdventHealth Waterman, Tavares, USA

**Keywords:** autoimmune disease, cryoglobulinemic vasculitis, diarrhea, gastrointestinal symptoms, hepatitis c virus (hcv), membranoproliferative glomerulonephritis, mixed cryoglobulinemia (mc), peripheral neuropathy, purpuric skin rashes, systemic vasculitis

## Abstract

This case displays a rare and complex manifestation of mixed cryoglobulinemia (MC) associated with chronic hepatitis C (HCV) infection, emphasizing the unusual gastrointestinal (GI) symptoms seen in this condition. While GI involvement is uncommon in cryoglobulinemia, it becomes crucial to consider when patients present with unexplained diarrhea, weight loss, and systemic vasculitis signs. Our patient exhibited persistent diarrhea, purpuric skin rashes, weight loss, fatigue, peripheral neuropathy, and renal dysfunction, all of which were suggestive of cryoglobulinemic vasculitis with renal involvement. Diagnosis was confirmed through cryoglobulin testing, reduced complement levels, elevated rheumatoid factor, and kidney biopsy results showing membranoproliferative glomerulonephritis. The case highlights the importance of recognizing and addressing otherwise unexplained GI symptoms in the diagnosis and management of cryoglobulinemia, particularly in the context of HCV infection. It also emphasizes the value of a multidisciplinary approach involving nephrology, rheumatology, dermatology, and gastroenterology in the management of this complex disease.

## Introduction

Cryoprecipitation refers to the precipitation of blood proteins when the temperature drops below 37°C [1]. The primary forms of cryoprecipitates are cryoglobulins, which precipitate from both plasma and serum, and cryofibrinogen, which precipitates only from plasma. Cryoglobulins are made up of immunoglobulins and sometimes complement components and can deposit in small- to medium-sized blood vessels [1]. This can lead to endothelial damage and dysfunction of organs. This deposition can lead to systemic symptoms such as pain in the joints, skin rashes, and kidney problems due to inflammatory reactions and blood flow that is impaired. Intestinal involvement in cryoglobulinemia is rare and can mimic the presentation of many other GI disease that present with chronic diarrhea. This case report illustrates an unusual case of cryoglobulinemia presenting with gastrointestinal symptoms of persistent diarrhea.

Cryoglobulinemia should be suspected in patients presenting with multiple systemic symptoms like joint pain, skin rashes, glomerulonephritis, or peripheral neuropathy [1]. It is more frequently exhibited in individuals that also present with chronic viral hepatitis (particularly hepatitis C virus (HCV)), monoclonal gammopathies (such as multiple myeloma, Waldenström macroglobulinemia, or the progression from monoclonal gammopathy of undetermined significance (MGUS) to monoclonal gammopathy of clinical significance (MGCS)), or connective tissue diseases (e.g., systemic lupus erythematosus (SLE) or Sjögren's syndrome) [1].

Over the past decade, it has become much more apparent that most individuals with essential mixed cryoglobulinemia (MC) are chronically infected with HCV. HCV is a small, single-stranded RNA virus that affects around 170 million people in the world, including around 3.2 million just in the United States [1]. Chronic HCV infection leads to progressive liver disease, which ranges from chronic hepatitis to cirrhosis and liver failure, ultimately lending the possibility of hepatocellular carcinoma. While much attention has traditionally been given to complications of chronic HCV relating to the liver, there is increasing recognition of notable extrahepatic manifestations like MC in some patients.

MC is distinguished by cryoglobulins containing multiple immunoglobulin components, such as IgM rheumatoid factor (RF) and polyclonal IgG [1]. This illness can be idiopathic or linked to autoimmune diseases, malignancies, or infections. It is divided into type 1, type II and type III [1].

Classification of cryoglobulinemia 

The Brouet criteria classifies cryoglobulinemia into three subtypes based on immunoglobulin composition: 

Type I 

It is characterized by monoclonal immunoglobulins, usually IgG or IgM, and is frequently associated with lymphoproliferative or hematologic disorders of B-cell origin, such as multiple myeloma, Waldenström macroglobulinemia, chronic lymphocytic leukemia, or monoclonal gammopathies like MGUS [2].

Type II

This form of MC involves a combination of monoclonal and polyclonal immunoglobulins. It is primarily linked with autoimmune diseases such as SLE, Sjogren’s syndrome and adult-onset Still disease, malignancies, lymphoproliferative disorders, vaccinations, and infections, like hepatitis B virus (HBV) infection, hepatitis C virus (HCV) infection [2], and HIV. Type II cryoglobulins typically consist of monoclonal IgM (or occasionally IgG or IgA) with rheumatoid factor (RF) activity, paired with polyclonal immunoglobulins [2].

Type III

This form of MC also involves polyclonal immunoglobulins, but unlike type II, there is no monoclonal component. Like type II, type III is linked to autoimmune diseases, malignancies, and infections, especially hepatitis C virus (HCV) infection [2].

Epidemiology and risk factors

The most common risk factor for cryoglobulinemia is drug use especially through sharing needles and syringes, with HCV infection acting as the predominant cause of cryoglobulinemic vasculitis, accounting for about 90% of cases [2]. The formation of HCV-related IgG and IgM rheumatoid factor (RF) leads to immune complex formation and complement activation, resulting in blood vessel inflammation.

While clinically significant cryoglobulinemia is rare, with a prevalence estimated at one in 100,000, detectable levels of cryoglobulins without obvious symptoms of vasculitis are found in many individuals with chronic infections or inflammation [1]. Specifically, cryoglobulins can be found in 15-20% of patients with HIV, 15-25% of patients with connective tissue diseases, 40-65% of those infected with hepatitis C, and up to 64% of patients co-infected with HIV and hepatitis C [1]. The proportion of individuals with type I cryoglobulins varies widely, but typically accounts for 5-25% of cases, while types II and III make up 50% and 40%, respectively [1].

## Case presentation

We describe a case of 69-year-old male who presented to the emergency department complaining of diarrhea of three months duration. The patient described his diarrhea as loose, watery, small volume, foul smelling, and occurring three to four times per day. The patient denied any specific dietary triggers. No other associated symptoms including melena, abdominal pain, and hematochezia were reported. The patient also denied any sick contacts, recent travel, fever, and chills. The patient complained of lower extremity non-specific pain with some associated weakness and rash, which he noticed for the same duration (three months). He was recently seen in a dermatology clinic and was prescribed topical steroids for his rash that consisted of reddish-purple non blanching macules/papules. The patient is anorexic and noted an unintentional weight loss of 10 pounds. He was also complaining of fatigue and that his “toes turn blue on cold exposure," which demonstrates secondary Raynaud’s phenomenon and damage to the blood vessels. Past medical history was notable for chronic hepatic C that is untreated. Patients’ social history includes occasional alcohol use, as well as he is an ex-smoker who is unable to quantify pack years. The patient also has a remote history of IV drug abuse. Family history was noncontributory. Home medications include triamcinolone for skin rash.

**Figure 1:**
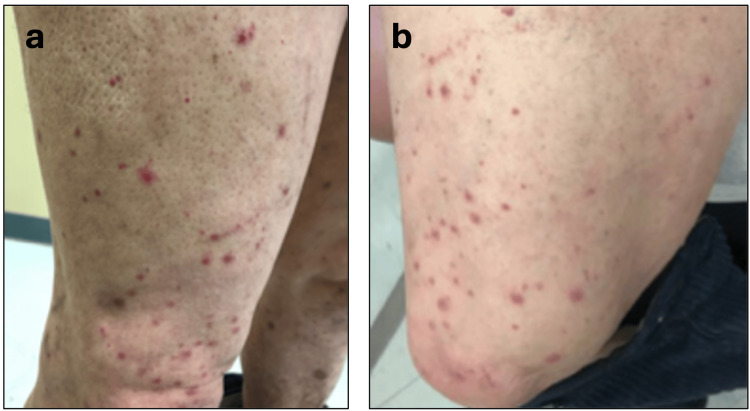
A: skin rash consisting of red/purple colored non-blanching maculopapular rash over lower extremities. b: a more magnified picture of the skin rash on the lower extremities.

His vitals on presentation were as follows: BP, 159/92 mmHg; pulse, 87; regular RR, 18; temp: 98.4F; and SPO_2_, 98% on room air. The rest of the physical exam remarkable for cachexia, splenomegaly, and skin rash consisting of red/purple colored non-blanching maculopapular rash over the abdomen and lower extremities (Figure 1). The neuro exam was non-focal. Cardiovascular and respiratory exams were within normal limits.

Hospital course

The patient was admitted for further workup/management and was started on supportive therapy and intravenous fluids. The patient was evaluated by nephrology, gastroenterology, rheumatology, and dermatology in view of multi-system involvement.

Acute kidney injury work up was done. Initial work-up was remarkable for elevated creatinine of 3.29 mg/dL, blood urea nitrogen (BUN) of 116 mg/dL, and estimated glomerular filtration rate (eGFR) of 18 ml/min/1.73 m^2^, consistent with severe renal insufficiency (Table 1). Further testing included urinalysis, which showed microscopic hematuria and a sub-nephrotic range proteinuria (Table 2). Complement levels (C3, C4, and total) were below normal range. Anti-nuclear factor (ANA), antineutrophil cytoplasmic antibody (C-ANCA), perinuclear anti-neutrophil cytoplasmic antibody 9P-ANCA (P-ANCA), anti-glomerular basement membrane (anti-GBM), anti-double-stranded DNA antibody (anti-dsDNA), and anti-cyclic citrullinated peptide (anti-CCP) antibodies were all negative (Table 3). C-reactive protein, sedimentation rate, and rheumatoid factors were positive, and the platelets were low (Table 4).

**Table 1:** Comprehensive metabolic panel (CMP)

Comprehensive metabolic panel (CMP)		Reference values
Blood urea nitrogen (BUN)	116 mg/dL	7-18 mg/dL
Creatinine (Cr)	3.29 mg/dL	0.6-1.2 mg/dL
Estimated glomerular filtration rate (eGFR)	18 ml/min/1.73m2	>90ml/min/1.73m2
Sodium (Na)	134 meq/L	136-146 mEq/L
Potassium (K)	5.8 meq/L	3.5-5.0 mEq/L
Chloride (Cl)	102 meq/L	95-105 mEq/L
Bicarbonate (HCO_3_)	17 meq/L	22-28 mEq/L
Calcium (Ca)	8.5 mg/dL	8.4-10.2 mg/dL
AG	15	>12mEq/L
Glucose	139 mg/dL.	70-110 mg/dL
Total proteins	5.3 g/dL	6.0-7.8 g/dL
Albumin	3.2 g/dL	3.5-5.5 g/dL
Total bilirubin	0.3 mg/dL.	0.1-1.0 mg/dL
Alkaline phosphatase (ALP)	64 U/L	25-100 U/L
Alanine transaminase (ALT)	15 U/L	10-40 U/L
Aspartate transaminase (AST)	18 U/L	12-38 U/L

**Table 2:** Urinalysis findings demonstrating hematuria and proteinuria in the patient.

Urinalysis (UA)		Reference values
pH	5.5	4.5-8
Color	Yellow	Colorless/pale yellow
Specific gravity (SG)	1.019	1.001-1.040
Blood	Large	0
Proteins	2	0
Bacteria	Few	0
Nitrite	Negative	0
Leukocyte esterase (LE)	Small	0
Red blood cell (RBC)	50-100/hpf	0
White blood cell (WBC)	5-10/hpf	0
Fine granular casts	2-5/hpf	0

**Table 3:** Autoimmune and infectious workup for vasculitis diagnosis

Advanced testing		Reference values
Complement levels C3	2	13-39 mg/dL
Complement levels C4	39	81-157 mg/dL
Complement levels total	< 14	42-95 U/mL
Erythrocyte sedimentation rate (ESR)	115 mm/hr	0-15 mm/h
Anti-GBM antibodies	Negative	Negative
Antinuclear antibody (ANA)	Negative	Negative
Rheumatoid factor (RF)	284	0-13 IU/mL
Cytoplasmic antineutrophil cytoplasmic antibody (C-ANCA), perinuclear antineutrophil cytoplasmic antibody (P-ANCA)	Negative	Negative
Cryoglobulin qualitative	Positive	Negative
Cryocrit	Not available	Negative
Anti-dsDNA	<12	Negative
Anti-CCP Ab	<8	Negative
Hepatitis C RNA viral load	5156 IU/mL.	0
Hepatitis C Ab	Reactive	Non-reactive

**Table 4:** Complete blood count (CBC)

Complete blood count (CBC)		Reference values
Hemoglobin	7.8 g/dL	13.5-17.5 g/dL
Hematocrit (HCT)	25.90%	41%-53%
Mean cell volume (MCV)	82fL	80-100 μm3
Platelets	140,000/uL	150,000-400,000/mm3
White blood cells	3700/ uL	4500-11,000/mm3
Neutrophils	77%	54-62%
Eosinophils	1.10%	1-3%
Lymphocytes	16%	25-33%
Monocytes	3.80%	3-7%
Immature granulocytes	0.80%	0-2%

**Figure 2:**
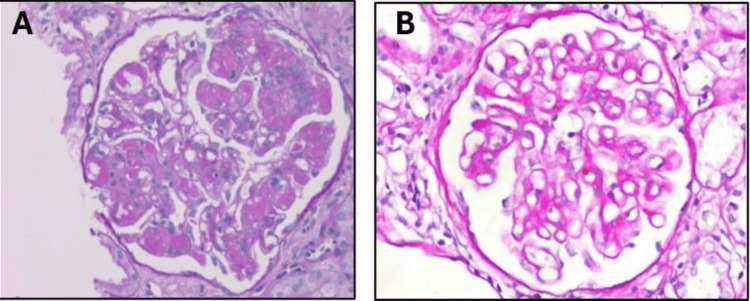
A. Membranoproliferative pattern with strongly PAS (Periodic acid Schiff)-positive cryoglobins plugs. B. Normal glomerulus under light microscopy (for comparison).

Given the nephritic picture, multisystem involvement, and history of untreated chronic hepatitis C, the cryoglobulin level was sent, which came back strongly positive. Hence, a kidney biopsy was pursued to confirm the diagnosis. The biopsy showed a membranoproliferative pattern in the glomeruli and strongly PAS-positive intracapillary cryoglobulin plugs by light microscopy and positive staining for IgG and IgM by immunofluorescence (Figure 2). In this clinical setting of the patient having a history of hepatitis C infection and positive cryoglobulinemia, these findings are diagnostic for cryoglobulinemic glomerulonephritis with multisystem involvement.

The patient also exhibited a right foot drop, significant for mononeuritis multiplex in which an EMG was performed, indicating peripheral neuropathy.

Given the predominant symptom of chronic diarrhea, stools studies were sent, which were negative for any leukocytes, fecal calprotectin, ova/parasites, giardia antigen, C-diff, and other viral PCR, essentially ruling out infectious and local inflammatory GI causes (Table 5). Hyperthyroidism was also ruled out with normal serum TSH levels. The quantitative hepatitis C viral load was 5156 IU/mL. Colonoscopy was deferred as the patient was very sick. Intestinal tuberculosis was ruled out with a negative QuantiFERON testing in view of immigrant history.

**Table 5:** Stool studies and additional testing

Stool studies and additional testing		Reference values
Occult blood stool	Negative	Negative
Fecal leukocytes	Negative	Negative
Fecal calprotectin	Negative	Negative
Stool ova and parasites	Negative	Negative
Stool culture	Negative	Negative
Stool Giardia antigen	Negative	Negative
Stool C-Diff PCR	Not detected	Not detected
Serum procalcitonin	0.53	0.02-0.10 ng/ dL
TSH	1.99 uIU/mL	0.4-4.0 μU/mL
Quantiferon:	Negative	Negative
AFB sputum culture	Negative x 3	Negative
MTB PCR	Not detected	Not detected
COVID-19 PCR	Not detected	Not detected
Repeat COVID-19 PCR	Not detected	Not detected
C-reactive protein	96.6 mg/dL	0-5.0 mg/dL
Transglutaminase Ab IgA	Negative	Negative
Strongyloides Ab	Negative	Negative
Blood cultures	Negative	Negative

Biopsy of the skin lesion was performed, which showed leukocytoclastic vasculitis. After confirmation of diagnosis, the patient was started on high-dose intravenous steroids by rheumatology. In addition to intravenous steroids, plasmapheresis was also recommended. However, the patient's course was complicated by severe *Clostridioides difficile* colitis and acute hypoxic respiratory failure secondary to alveolar hemorrhage (confirmed with bronchoscopy and BAL), another manifestation of cryoglobulinemic vasculitis.

Despite aggressive management, the patient developed multiorgan failure and succumbed to the disease.

## Discussion

Persistent immune stimulation and lymphoproliferation lead to a heightened production of mono-, oligo-, or polyclonal immunoglobulins, which may form cryoglobulins. These cryoglobulins circulate in the bloodstream and may precipitate, forming immune complexes that deposit in small- to medium-sized blood vessels [2]. This leads to inflammation and obstruction of vessels. The deposition of immune complexes triggers an inflammatory response, causing endothelial damage and immune cell attraction of lymphocytes and macrophages. Activation of the complement system further escalates the inflammation and tissue damage. The most commonly affected organs include the skin, kidneys, and peripheral nerves, leading to symptoms such as purpura, glomerulonephritis, and neuropathy [2].

Clinical manifestations

Type I Cryoglobulinemia

This type is characterized by vascular symptoms such as ischemia, livedo reticularis (a lace-like erythematous pattern that blanches with pressure), and skin necrosis. In particular, 70-85% of patients present with skin manifestations including but not limited to ulcers and necrosis [2]. Other frequent symptoms include peripheral neuropathy, arthralgia, and arthritis, but central nervous system (CNS), pulmonary, cardiac, or gastrointestinal involvement is rare [2].

Type II/III (Mixed) Cryoglobulinemia

This type generally manifests with arthralgia, fatigue, and myalgia. Palpable purpura, associated with vasculitis, and sensory changes from peripheral neuropathy are common [2]. The "Meltzer triad," which includes purpura, arthralgia, and weakness, was seen in our patient. Purpura, typically found on the legs, is the most frequent symptom and can extend to the torso and upper limbs. Our patient exhibited a skin rash on the lower extremities that consisted of reddish purple non blanching macules/papules in which topical steroids was initiated [3].

Our patient also exhibited a right foot drop, significant for mononeuritis multiplex in which an EMG was performed, indicating peripheral neuropathy.

Our patient’s social history indicated that he was a chronic IV drug abuser. In addition to the clinical picture our patient exhibited, his Hep C RNA viral load was 5,156 IU/mL, and HepC antibody was positive, indicating a diagnosis of chronic HCV. HCV is tightly linked with type II/III MC presentation.

A prevailing theory for HCV-related cryoglobulinemia is that chronic antigenic stimulation results in clonal expansion of B-lymphocytes [4]. Additional methods include a t(14:18) chromosomal translocation in HCV-infected individuals, activating Bcl-2, which facilitates B-cell survival and increases autoantibody and cryoglobulin production [4]. The HCV E2 protein binds CD81 on B cells, lowering activation thresholds and increasing antigen-reactive B cells [4]. In addition, molecular mimicry by HCV proteins like NS5A may activate B-lymphocytes. B-cell activating factor (BAFF) might also be a factor in the condition [4]. The expansion of rheumatoid factor-producing B cells, characteristic of HCV-associated cryoglobulinemia, indicates a pathogenic role of these autoantibodies exhibited by our patients increased rheumatoid factor.

Laboratory and diagnostic findings

Although MC can be diagnosed clinically, the main laboratory findings in cryoglobulinemia include detectable cryoglobulins (cryocrit) and a low C4 complement level. Advanced testing of our patient shows his C3, C4, and total complement levels to be below normal range. Cryocrit levels are not available. However, cryoglobulins' qualitative testing was positive. Upon review of our patient’s clinical manifestations, labs, and comorbidities, the diagnosis of type II/III (mixed) cryoglobulinemia was made. Biopsies of affected organs are not always required for diagnosis, but they can offer valuable histopathologic evidence of cryoglobulinemic vasculitis. Our patient did undergo a kidney biopsy, which on immunofluorescence microscopy did show membranoproliferative glomerulonephritis. This is seen in 60-80% of cases and is the most common pattern [2]. Endocapillary proliferation was seen along with deposits of cryoglobulins, immunoglobulins, and complement proteins in subendothelial locations. Periodic acid Sciff (PAS) stain was performed and demonstrated positive staining of the eosinophilic deposits, indicating cryoglobulinemic deposits. These findings were diagnostic for cryoglobulinemic glomerulonephritis. Type I membranoproliferative glomerulonephritis is predominantly associated with HCV infection. This involves glomerular inflammation, upon which the filtration barrier to RBC and protein is lost and GFR decreases as renal failure persists. This loss of RBC and protein in the urine can be seen in the patient’s urinalysis. The patient’s eGFR was calculated to be 18.1 ml/min/1.73 m^2^, which is considered stage 3 acute kidney injury. This decreased GFR can lead to an increased BUN/Cr ratio and increased renin release, manifesting as hypertension, which was also exhibited in our patient’s blood pressure readings as our patient is a stage 2 hypertensive.

The occurrence of diarrhea alongside MC is an uncommon but noteworthy clinical presentation. The primary clinical feature of systemic vasculitis affecting the gastrointestinal tract is usually attributed to mesenteric ischemia and/or ischemic colitis, which can lead to infarction [5]. Patients commonly report abdominal pain, which may be either acute or chronic. Chronic abdominal pain is also frequently observed in chronic mesenteric ischemia caused by a low-flow state, typically accompanied by postprandial pain, weight loss, nausea, vomiting, and diarrhea [5]. Our patient, for example, experienced weight loss and chronic diarrhea. In cases where systemic vasculitis is diagnosed, gastrointestinal vasculitis must be taken into account in patients presenting with signs of gastrointestinal ischemia and elevated inflammation markers such as erythrocyte sedimentation rate (ESR) and C-reactive protein (CRP), especially in the absence of other risk factors for atherosclerotic vascular disease. For those with a known diagnosis of vasculitis, the focus of evaluation is to exclude other causes of gastrointestinal symptoms, followed by medical intervention targeting the underlying vasculitis.

Treatment

The treatment of cryoglobulinemia is dependent on the underlying cause, symptom severity, and organ involvement. For MC, treatment targets the underlying autoimmune or infectious condition. In essential MC, which in most situations has a more severe clinical course, therapy includes steroids combined with rituximab, with steroids tapered over time [2]. Plasmapheresis and immunosuppressive therapies, including glucocorticoids and rituximab, could be employed for quickly advancing or life-threatening cases [2]. Addressing the underlying infection or autoimmune disorder is crucial. 

HCV infections are the primary cause of MC, and the initiation of direct antiviral therapies has altered the therapeutic focus toward pan-genotypic antiviral regimens (e.g., sofosbuvir or velpatasvir with glecaprevir or pibrentasvir) [2]. Although interferon (IFN) was previously used, these direct antiviral agents are better suited and less harmful. Cyclophosphamide may be used in conjunction with apheresis for high cryocrit levels, although its application has declined with the rise of rituximab [2].

Our patient received high-dose IV steroids, but our patient’s hospital course got complicated by multi-organ dysfunction, and due to rapid clinical deterioration, the patient was not able to receive other standard therapies and, unfortunately, passed away.

## Conclusions

This case illustrates the complex presentation of MC associated with chronic HCV infection, highlighting the multifaceted nature of the disease and its rare gastrointestinal manifestations. While gastrointestinal symptoms in cryoglobulinemia are uncommon, they should be addressed when a patient presents with unexplained diarrhea, weight loss, and systemic signs of vasculitis. Our patient's presentation was marked by persistent diarrhea, purpuric skin rashes, weight loss, fatigue, peripheral neuropathy, and kidney dysfunction, all of which pointed to cryoglobulinemic vasculitis with renal involvement, as confirmed by biopsy showing membranoproliferative glomerulonephritis. The association between HCV infection and MC is well-established, with continuous antigenic stimulation leading to the generation of cryoglobulins that deposit in small- to medium-sized blood vessels, contributing to systemic vasculitis. The diagnosis of cryoglobulinemia was supported by positive cryoglobulin testing, decreased complement levels, and a markedly elevated rheumatoid factor. In addition, the renal biopsy findings further validated the diagnosis of cryoglobulinemic glomerulonephritis, while the patient's elevated blood urea nitrogen and creatinine levels, along with sub-nephrotic-range proteinuria, pointed to significant kidney involvement. Unfortunately, due to the patients rapid clinical deterioration, the patient was not able to complete standard therapy. The development of a *Clostridium difficile* infection highlights the risks associated with immunosuppressive therapy and emphasizes the importance of careful monitoring during treatment. The case also stresses the critical role of interdisciplinary collaboration, including nephrology, rheumatology, dermatology, and gastroenterology, in the comprehensive management of such complex cases. In conclusion, this case points out the importance of recognizing the diverse clinical presentations of MC, particularly in patients with chronic HCV infection. Timely identification and treatment are essential in preventing irreversible organ damage, and a multidisciplinary approach is fundamental for dealing with the varied manifestations of this challenging condition.
